# Toxicant Exposure and Bioaccumulation: A Common and Potentially Reversible Cause of Cognitive Dysfunction and Dementia

**DOI:** 10.1155/2015/620143

**Published:** 2015-02-04

**Authors:** Stephen J. Genuis, Kasie L. Kelln

**Affiliations:** ^1^Faculty of Medicine at the University of Calgary, Calgary, AB, Canada T2N 4N1; ^2^Faculty of Medicine at the University of Saskatchewan, Saskatoon, SK, Canada S7N 5E5

## Abstract

Juxtaposed alongside the ongoing rise in the incidence and prevalence of dementia, is the surge of recent research confirming widespread exposure and bioaccumulation of chemical toxicants. Evidence from sources such as the Centers for Disease Control reveals that most people have accrued varying degrees of assorted toxic pollutants including heavy metals, flame retardants, and pesticide residues within their bodies. It has been well established that many of these toxicants have neurodegenerative as well as neurodevelopmental impact as a result of various pathophysiologic mechanisms including neuronal mitochondrial toxicity and disruption of neurotransmitter regulation. Elimination of stockpiled toxicants from the body may diminish adverse toxicant impact on human biology and allow restoration of normal physiological function. Incorporating a review of medical literature on toxicant exposure and dementia with a case history of a lead-exposed individual diagnosed with dementia, this paper will discuss a much overlooked and potentially widespread cause of declining brain function and dementia.

## 1. Introduction

The rising incidence and prevalence of dementia has become an enormous public health challenge. At the 2009 International Conference on Alzheimer's disease in Vienna, Dr. William Thies, Chief Medical and Scientific Officer for the Alzheimer's Association, summarized the challenge with his comment: “The number of people affected by Alzheimer's and dementia is growing at an epidemic pace, and the skyrocketing financial and personal costs will devastate the world's economies and health care systems, and far too many families” [1].

A diagnosis of dementia has profound personal and social implications for patients and their loved ones. The most common type of dementia, Alzheimer's disease (AD), occurs in about one in every nine people aged 65 and over, with a continued rise in incidence anticipated in the coming years [2]. Dementia is associated with high rates of morbidity and mortality and is accompanied by enormous personal suffering for patients, as well as challenging management problems for families, physicians, and health care systems [3]. It appears that efforts at prevention and treatment of dementia for the last two decades have had little effect on the rising prevalence of this health problem or the quality of life for many patients suffering with this disorder [3]. By integrating information from the medical and scientific literature with clinical outcomes from a case history of an individual diagnosed with dementia, this paper will discuss toxicant exposure and bioaccumulation, a much overlooked and potentially modifiable cause of declining brain function and dementia.

## 2. Sources of Information

This review was prepared by assessing medical and scientific literature from MEDLINE/PubMed, several books and toxicology journals, conference proceedings, government publications, and environmental health periodicals. References cited in identified publications were examined for additional relevant writings. Searching techniques included keyword searches with terms related to chemical exposure and dementia, toxicants and AD (Alzheimer's disease), pollutants and neurodegenerative disease, environmental health sciences and the elderly, neurotoxicity with poisoning, and detoxification.

A primary observation was that limited scientific literature exists on the link between neurodegenerative processes and toxicant bioaccumulation, on the pathogenesis involved, as well as the management of degenerative states associated with toxicant accrual. The first author's professional observations and published research as an environmental health physician were also incorporated into the discussion of management strategies. The format of an integrated clinical review was chosen as such reviews play a pivotal role in scientific research and professional practice in emerging medical issues with limited primary study and uncharted clinical territory [4].

## 3. Background

Recent evidence has emerged linking the development of dementia with various inorganic and organic toxicants in our environment [5–9]. For example, a recent report from* JAMA Neurology* found that elevated serum levels of the toxicant DDE (dichlorodiphenyldichloroethylene), a direct reflection of brain DDE levels [10], are associated with increased susceptibility for developing Alzheimer's disease [10]. DDE is a persistent metabolite of the organochlorine compound DDT (dichlorodiphenyltrichloroethane), a pesticide widely used following World War II. DDT was banned in most countries in the early 1970's because of persistence within the human organism and the environment and the consequent adverse health effects associated with exposure and bioaccumulation [11]. Metabolic byproducts of DDT such as DDE continue to be found in serum samples of 75–80% of the population due to its long half life and continued exposure. People remain at risk of exposure by consuming imported foods from countries where DDT is still used as well as from pollution in soil and water sources [11, 12].

A growing body of evidence has recently implicated many other exposures to organic chemicals as potential determinants in the development of dementia (Table 1). Formaldehyde, a common indoor air pollutant off-gassing from wood products and various other sources within the home, has been linked to dementia [13–15] Exposure to methanol, a potential contaminant of ambient air and some foodstuffs, has also been associated with neurodegeneration in animals [16, 17]. Some common pharmaceutical exposures including ongoing use of benzodiazepines have been linked to an increased risk of Alzheimer's disease [18]. Various common components of air pollution, a widespread problem in many areas of the world, have also been implicated in the escalating pandemic of dementia [19].

Many other classes of common bioaccumulative organic compounds have also been recognized to have neurotoxic effects including brominated flame retardants (PBDEs) [20], solvents [21], “nonstick” perfluorinated compounds [22], polychlorinated biphenyls (PCBs) [23], and various types of pesticides [7, 24]. With further research, it remains to be seen which organic pollutants alone or in combination will be significant determinants of the growing epidemic of dementia.

As well as organic compounds, the scientific literature highlights the myriad adverse effects of various inorganic toxic elements such as mercury [25–27], aluminum [7, 8, 28], arsenic [8, 27, 29], copper [30–33], and particularly lead [7, 34]. Elevated levels of lead have been implicated in hypertension and linked to vascular causes of memory impairment and dementia [35, 36]. The acquisition, storage, and retrieval of verbal information also appears to be affected by cumulative exposure to lead [37]. After years of research on the effects of lead, Stewart and Schwartz suggest the following: “a significant proportion of what is considered to be normal age-related cognitive decline may, in fact, be due to past exposure to neurotoxicants such as lead” [38]. While lead is an established neurotoxicant that is associated with neurodevelopmental deficits in children, recent evidence suggests that even low levels of ambient lead exposure in the air also appears to increase the risk of neurodegenerative disease [6]. 

*(i) Toxicant Exposure and Bioaccumulation.* The problem of toxicant exposure and bioaccumulation in the population appears to be rising rapidly, yet this escalating health threat remains insufficiently recognized by many within the medical community. A recent study by the Centers for Disease Control reveals that most American adults and children of both genders have bioaccumulated numerous toxic pollutants in their body [39]. A similar epidemiological study in Canada also reveals a widespread accrual of assorted toxicants [40]. Furthermore, a recent cord-blood study on infants revealed that at birth, most neonates have already accumulated assorted toxicants reflecting maternal exposure [41]. The neurodevelopmental sequelae of such exposures can be observed in the swelling pandemic of autism [42] and other neurodevelopmental disorders [43, 44]. Yet, while most people are aware of the risks associated with toxicant exposure and accrual from pollutants such as nicotine and cadmium found in cigarettes, many are not yet apprised of the adverse outcomes of exposures to various other chemical agents that are at least as harmful as what is found in tobacco.

Groups such as the Pediatric Academic Societies have begun to speak out regarding pediatric toxicant exposure announcing that “low level exposure to environmental toxicity may be impacting the functioning of the current generation” [45]. However, there has been little appreciation within the medical community of neurodegenerative toxicants and their potential impact on the maturing adult segment of the population. As well as dementia, research continues to elucidate the relationship between environmental toxicant exposure and the development of other neurodegenerative diseases including Parkinson's Disease, Multiple Sclerosis, and Amyotrophic Lateral Sclerosis [7, 28, 30, 46–48]. Table 1 provides an overview of some of the chemical toxicants that have been directly associated with neurodegenerative disease.

Many assume that, in the absence of ongoing exposure, toxicants are eliminated efficiently. It has recently been recognized, however, that many toxicants including some solvents, pesticides, petrochemicals, mycotoxins, and assorted synthetic chemical agents are persistent pollutants with half-lives that can last for many years or even decades [49–51]. Accordingly, the problem of neural decline may be the result of exposures that occurred long ago. With stockpiled toxicants persistently disrupting normal human processes and physiology, it is understandable that the resulting pathophysiology may induce chronic disease.

Some challenge the idea that apparently miniscule doses of toxicant exposures can have such a profound impact on physiological function; there are two important considerations in response to this misguided claim. First, toxicant exposures do not usually occur as single isolated events. Many people, through occupation or habitat environments, are repeatedly exposed to chemical toxicants and are therefore accruing persistent pollutants on an ongoing basis, some of which will remain in tissues for decades. This is of particular note with the potential degree of exposure via polluted air, as the average adult inhales about 10 thousand liters of air per day. Whereas single exposures may be less problematic and perhaps harmless depending on the particular toxic agent and the dose, the repeated exposure to toxic agents presents an accumulated burden or total load to the intricate physiological functioning of the body [50].

Second, it is noteworthy that standard biochemicals within our inherent physiology, as well as prescribed pharmaceutical agents, are bioactive at levels of parts per billion (ppb) and some at parts per trillion (ppt). For example, ethinyl estradiol in the birth control pill maintains contraceptive efficacy at serum levels below 62 to 83 ppt (parts per trillion) [52]. It is hardly surprising, therefore, that serum concentrations of various toxicant chemicals often measured in ppb (one thousand times higher than ppt), or parts per million, (an order of magnitude one million times higher than ppt), might have biological impact on the human organism. In review, various toxicants can have extremely potent physiological impact at seemingly miniscule doses [53], with incremental impact for some pollutants at increasing levels of accrual [54, 55].

*(ii) Toxicant Mechanisms of Harm.* Environmental pollutants may disrupt biological function through various destructive pathways. Pathophysiological mechanisms of harm include mitochondrial damage [56], oxidative stress [57], cell death [58], neurotransmitter dysregulation [56], endocrine disruption [59], and epigenetic modification [60]. Toxicant induced mitochondrial dysfunction appears to be a primary mechanism of harm in neurodegenerative diseases such as AD [7]. Oxidative stress is also an early contributor to neurodegeneration as markers of oxidative stress have been found in neurons of AD patients as well as those with mild cognitive impairment [7]. Toxicant induced cell death via apoptosis, autophagy, and necrosis may initiate or exacerbate the neurodegenerative process [61]. Cell death, for example, is a known consequence of accrual of PBDEs [20].

As plaques formed by deposition of the beta-amyloid peptide within the brain have been routinely observed in postmortem assessments of AD patients [62], it has been hypothesized that Alzheimer's' dementia may be the result of amyloid plaque buildup around neurons, often resulting in cell apoptosis. It has been recently recognized, however, that development of such peptide plaques is often the result of exposure and bioaccumulation of toxicants such as PBDEs [20]. As the brain contains considerable amounts of fatty tissue, this organ accrues lipophilic compounds such as PBDEs which are among the most common toxicant exposures since PBDEs have been used extensively to reduce flammability in everyday consumer products such as mattresses and furniture (which then off-gas and contaminate individuals).

There is emerging evidence that lead, in particular, impairs cell-to-cell communication with neurotransmitters such as acetylcholine and glutamate by blocking neuronal calcium and potassium channels [26]. Rat models have shown that lead exposure specifically affects the hippocampus by decreasing potassium stimulation and release of glutamate and GABA [63]. Lead has also been shown to increase the permeability of the blood brain barrier leaving the brain vulnerable to a variety of other insults [26]. Alterations in the blood brain barrier have been implicated in neurodegenerative diseases including Parkinson's Disease, Amyotrophic Lateral Sclerosis, and Alzheimer's Dementia [7].

Many toxicants are known endocrine disruptors: some of the most notable disruptors are pesticides such as DDE [64] and plasticizers such as Bisphenol A and phthalates [59]. These latter toxicants have also been implicated in neurotoxicity as a result of their hormonal impact on neurological function [59]. Moreover, it has been suggested that epigenetic alterations resulting from histone modification from exposure to these plasticizers may be a causative mechanism in toxicant induced neurodegeneration [46]. Furthermore, toxicant induced epigenetic modification has recently been recognized to have transgenerational effects that may impact future generations [60]. In review, through assorted pathophysiological mechanisms, toxicants have become a ubiquitous but poorly recognized cause of ill-health in the 21st century [65].

*(iii) Potential Treatments.* With recent recognition that various pollutants are neurotoxicants, there is emerging consideration of outcomes that might be achieved if measures are taken to eliminate the toxicant burden [66]. Recent research has highlighted assorted interventions that can be used to eliminate accrued persistent pollutants [67–74]. Evidence of neuropsychiatric improvement has been observed with individuals receiving treatment to facilitate toxicant elimination of retained pollutants such as lead [70] and mercury [75]. Preliminary evidence also suggests significant amelioration of morbidity following interventions to clear toxicants in autistic children contaminated with inorganic pollutants [76, 77]. Assorted other medical states have improved following treatment to eliminate persistent toxicants from the body [67, 68], Bredesen reports on the reversal of cognitive decline through a personalized therapeutic program which incorporates the exclusion of heavy metal toxicity in patients with AD [78]. Lowering serum free copper with zinc therapy has been shown to protect against memory impairment in older adults [31] and Squitti et al. further advocate that using low-copper diets may help prevent neurodegenerative memory impairment associated with Alzheimer's disease [32, 33].

These findings have enormous clinical significance for patients suffering with dementia. As the development of dementia may follow environmental exposure to and accrual of chemical toxicants, a potential approach for patients with dementia might involve assessing for and eliminating stockpiled toxicants. The following case history demonstrates how investigating for and then addressing the underlying toxicant burden improved the quality of life and cognitive function of a patient with dementia.

## 4. Case History

A 69-year-old married, retired male with complaints of diminishing cognitive function, severe memory impairment, and profound fatigue was brought to an environmental health physician by his wife and daughter (a nurse). The patient had seen a neurologist and following the clinical assessment, an MRI and a CT scan, a diagnosis of dementia was given. There was no history of brain injury, seizures, stroke, or substance abuse.

The family indicated that the patient moved to Canada from Europe in excellent health in his midtwenties and took a job in a glass shop making stained-glass windows. Soon after commencing this work, which involved the heating of metal rims in a confined space with inadequate ventilation, the patient began experiencing progressive mental health problems and was diagnosed with bipolar disorder. Although he left his job making stained-glass windows, he continued to suffer with recurrent episodes of depression and manic episodes for the next 35 years. Under the care of a psychiatrist, he had been treated with lithium for 25 years, as well as with antidepressants and mood stabilizers for the bipolar illness.

Over the last five years, however, the patient displayed increasing cognitive decline, with escalating memory impairment and social isolation. He had become increasingly dependent on his family for care and support. By the time he presented to the environmental health clinic, he barely spoke and was not attentive to discussions about his health. With a diagnosis of irreversible dementia, discussion ensued between family and caregivers about potential placement in an extended care facility for the remainder of his life. His medications at the time of presentation included Quetiapine and Divalproex.

Other than signs of profound cognitive decline, physical examination did not reveal any objective findings except a chronic fungal infection of the patient's toenails. Various investigations were undertaken as part of the assessment at the environmental health facility. Noteworthy laboratory results after provocation testing revealed a significant burden of the toxic metal lead, most likely from exposure while heating lead-containing metal rims in an inadequately ventilated room as part of the process of making stained-glass windows in his previous employment. His tests also revealed elevated ferritin, immunoglobulin E, C-reactive protein, and positive antinuclear antibody, all common findings in patients with toxicant bioaccumulation. As well, his creatinine was elevated and glomerular filtration rate was decreased, likely the result of years of lithium use.

The family was intrigued by the evidence for lead accumulation. They presented the information to the psychiatrist who refused to believe there was evidence of lead accrual. Disparaging in his comments, he suggested that this was nonsense and indicated he would prove this by testing. He did a blood and urine level for lead and confirmed minimal amounts of lead on such testing. The family presented this information to the environmental health physician prior to commencing any treatment. As was explained to the family in detail, the body has different compartments. It is well recognized that lead and many other toxicants sequester in locations such as bone, brain, and adipose tissue and do not typically remain in the blood [25]. That is, there may be an absence of lead in blood or urine testing with considerable retained stores in tissues. Testing for lead accumulation within tissues requires provocation testing where stores are first mobilized, and agents such as DMSA or EDTA are then used to bind the lead and release it into urine and stool [79, 80].

With evidence of lead bioaccumulation, therapies to diminish the body burden of this toxic metal were commenced. Various toxicant elimination interventions including skin depuration [70], oral DMSA [81], and EDTA [82] by rectal suppository were used to get the lead out. Nutritional supplementation was provided to prevent mineral deficiency, a potential occurrence with treatments to eliminate toxic elements. The patient and his spouse were educated to make changes in their lives to preclude ongoing exposure to various potential toxicants.

Within six months, the patient noticed considerable improvement in memory and mood. Within one year of treatment, friends and family concluded he had made a dramatic recovery and observed a restoration of his distinctive sense of humor. The chronic fungal infection in the patient's toenails resolved spontaneously within 18 months of treatment. Repeated follow-up provocative metal testing revealed that excretion of lead had diminished considerably, likely reflecting a decline in the total body burden. Lab values of ferritin and immunoglobulin E subsequently returned to normal range within two years. The patient and his family were happy with the results, but when the patient felt well and was able to function, he became less compliant and eventually discontinued further treatment to eliminate the total burden of lead. Six years after his initial visit for environmental health assessment, the patient continues to feel well, now living independently with his spouse and enjoying a good quality of life.

## 5. Discussion

Cognitive decline associated with lead exposure has been recognized for centuries. The earliest known descriptions of lead poisoning in literature originate from the Greek physician and poet Nikander of Colophon circa 200 BC, with descriptions of health damage due to occupational exposure to this toxic metal [83]. Recent discussion has also considered the impact of lead exposure on some historical events. It has been hypothesized by some historians, for example, that the fall of the Roman Empire may have been influenced by an epidemic of lead exposure among Roman aristocracy due to the presence of this toxic element in pottery, utensils, water piping, and drinking vessels for wine [84]. Renowned artist Vincent Van Gogh was believed to have suffered lead toxicity from eating chips of lead-based paint while creating his well-known works of art [85], and, after researchers discovered lead deposited in his hair and bones [86], lead toxicity may have been a major determinant in the mental health and auditory afflictions suffered by the celebrated composer Ludwig van Beethoven.

While the effects of high levels of lead exposure are well understood, a growing body of literature has raised concern with very low levels of lead bioaccumulation as well [87, 88]. Manifestations of lead stockpiling may be silent for many years, and it is now known that even blood levels below 10 ug/dL can result in renal dysfunction, hypertension, and essential tremor [87]. Such low levels of lead were previously considered acceptable and safe; new evidence highlighting the adverse effects of very low levels of lead on human health has resulted in a significant shift downward in what is considered to be acceptable levels of exposure [83]. This recognition has recently been affirmed with the finding that even ambient lead exposure from air pollution has been associated with developing Alzheimer's disease as demonstrated by MRI imaging showing hippocampal and entorhinal cortex atrophy [89].

Although many public health initiatives have been undertaken to decrease toxic element exposure in the population, the bioaccumulation of lead and resulting health problems are still surprisingly prevalent and patients continue to be exposed from a variety of sources [87]. In fact, a recent study done by the CDC found that 13% of pregnant women had blood lead levels above acceptable limits [90], possibly related in some cases to exposure from contaminated prenatal supplements [91]. With a prolonged half-life, lead may persist in the body for many years even after absence of continued exposure [92]. 

*Neurodegenerative Disease and Toxicant Exposure.* As toxicants have the potential to greatly affect neurological function [7], the increasing rates of neurodegenerative diseases in the population may not be solely due to people surviving longer, but also due to the increasing toxicity from chemicals in our environment [29]. Air pollutants, particularly ozone, particulate matter, and nitrogen dioxide have been linked to cognitive decline in older adults living in Los Angeles [19]. Although not yet fully understood, it has been proposed that air pollution may contribute to brain inflammation, microglia activation, and white matter abnormalities [47]. While exposure to endocrine disrupting chemicals like brominated flame retardants, perfluorinated compounds, phthalates, and phenols during gestation or early life has been correlated with neurotoxic effects in children [93], active research is now exploring the impact of these toxicants on physiologic neurogenesis in older populations and the associated risk for neurodegeneration [59].

Given that the prevalence of dementia is expected to increase by 40 percent over the next decade, it may be clinically indicated to assess for toxicant bioaccumulation in patients presenting with cognitive decline. One of the main challenges with assessments for toxicant accrual with the central nervous system, however, is that serum measurements and even adipose tissue biopsies may underestimate and not be reflective of the burden of pollutants within other tissues such as brain [73, 94, 95]. Accordingly, techniques for mobilization of tissue-based toxicants such as caloric restriction [96], thermal therapy, and exercise may need to be incorporated into toxicant panel biomonitoring [42].

If toxicant bioaccumulation is evident, interventions should be undertaken to eliminate the toxicant burden. This may not only ameliorate symptoms of dementia and cognitive decline, but also may have a positive impact on a variety of other body systems and help prevent further neurodegeneration. Such interventions have the potential to greatly impact quality of life for both patients and their families. Since rates of hospitalization for patients with dementia are three times higher per year as compared to other older patients without dementia [2], the potential benefits to the health care system are also evident. 

*Medical Education.* As recent evidence suggests that 70–90% of chronic disease is likely related to environmental determinants [97], ample understanding of environmental health science is required for the practice of contemporary health care [98]. Although high dose acute poisoning may be easy to recognize and the effects of such exposures are well known, more subtle low dose and cumulative chronic exposures can be challenging to identify and clinical care requires knowledge and skills about environmental sources of toxicants [99]. While a call to incorporate environmental medicine into the medical curricula has been in the literature for some time [100], such education remains largely absent from medical school programs [99]. Many physicians are thus ill-equipped to provide environmental health services including toxicant assessment and elimination to patients presenting with diverse complaints including neurodegenerative illness [100].

Formalizing basic environmental medicine education as part of medical school curricula, specialty training, and continuing medical education for practitioners would help equip physicians with the knowledge and skills necessary to correctly identify varying toxicant exposures in patients. Such training would also allow health providers to incorporate evidence-based interventions to facilitate elimination of toxicants. Furthermore, this knowledge would allow physicians to identify patients at risk and provide education regarding prevention of exposure.

## 6. Conclusion

Exposure to and bioaccumulation of various chemical pollutants is increasingly being established as a widespread determinant in the development of neurodegenerative illness such as Alzheimer's Disease. Despite this recognition, chemical toxicant accrual as an etiological and potential reversible cause of illness has generally been overlooked in medicine, neurology, public health, and psychiatry [48, 101]. While most people recognize that high-dose exposure to various chemical agents can alter brain function, there is not yet widespread recognition (other than with cigarettes) that ongoing exposure to low levels of toxic pollutants and chronic bioaccumulationcan of these agents can cause sustained disruption of physiologic function including brain biology. In keeping with recent evidence that the majority of chronic illness is not primarily genetic, but environmental in origin [97], a lack of etiology-centered medicine is sometimes allowing the underlying causes of disease to go unnoticed while clinicians focus on symptomatic management [102].

Exposure to toxic substances has historically been recognized as one of the five fundamental etiologies of chronic illness [103]. Identification and eradication of underlying toxicant burdens can be life-changing for patients and their families [9]. Prior to his treatment and recovery, the patient discussed in the case history presented in this paper was being considered for transfer to a long-term care facility in order to meet his increasing needs for assistance. His remarkable recovery is now allowing him to live independently with his wife and to enjoy a productive life.

Despite expanding attention in major public health and toxicology journals to the field of toxicant exposures as determinants of chronic illness, there continues to be a constant stream of new potential pollutants being introduced into the environment. With inadequate scrutiny and regulation prior to release, there is often insufficient knowledge of the implications that such toxicants may have on individual and public health [29]. Increasing exploration of the potential impact of environmental toxicants on current and future generations must be a priority [26].

Our culture has increasingly equated advanced age with sickness and disability. If we continue to neglect the impact of a lifetime of environmental pollutants on aging populations, we may forego a future of healthy and capable older adults, as is evidenced by the expanding contemporary challenge of neurodegenerative illness. Taking a precautionary approach and having strategies for prevention of exposure is of paramount importance [26]. Furthermore, integrating toxicant etiology-centered approaches to illness may reveal the true underlying cause of the illness [103] and allow physicians to effectively treat or even reverse disease. The potential to limit or reverse a condition such as dementia might have considerable impact on individuals, families, and population groups.

Comprised of about sixty percent fat, a concentration higher than is found in any organ in healthy individuals, the human brain is a magnet for lipophilic persistent toxicants. In an age of unprecedented and ubiquitous chemical exposure and bioaccumulation, as confirmed by medical organizations such as the Centers for Disease Control [39] and government agencies such as Health Canada [40], it is recommended that all patients with inexplicable cognitive decline and dementia be investigated for evidence of accrued pollutants and treated when diagnosed to considerably diminish their toxicant burden [73].

## Figures and Tables

**Table 1:** Exposures potentially associated with neurodegenerative disease.

Metals	Lead [6–9, 25–27, 29, 35, 37, 38, 47, 48, 63, 83, 84, 87, 90, 92, 104]
	Mercury [8, 25–27]
	Aluminum [7, 8, 28]
	Zinc overload [7]
	Manganese [8, 29, 47]
	Arsenic [8, 27, 29]
	Tin [8]
	Copper [30–33]

Pesticides	Dichlorodiphenyldichloroethylene [10] (DDE)
	Aldrin [29]
	Chlordane [29]
	Heptachlor [29]
	Rotenone [5, 7]
	Dieldrin [5]
	Methyl parathion [26]
	Organophosphates [5, 7, 26, 46, 48]
	Maneb [5, 29]
	Paraquat [5, 7, 29, 46]
	Pyrethroids [5]

Flame retardants	
	Hexabromocyclododecane [20]
	Tetrabromobisphenol-A [20]
Brominated [93]	Decabromodiphenyl ether [20]
	6-Hydroxy-2,2′,4,4′-tetrabromodiphenyl ether [105]
Chlorinated	2,2′,4,4′-tetrachlorobiphenyl [105]
	Trichloroethylene [7]

	Carbon disulfide [26, 29]
Solvents [48]	Toluene [26]
	Perchloroethylene (PERC) [106]

Pharmaceuticals	Anesthetic agents [107]
	Benzodiazepines [18]

Air pollution [6, 47]	Particulate matter [19]
	Ozone [19]
	Nitrogen dioxide [19]
	Second hand smoke [27]
	Carbon monoxide [8, 48]

Plasticizers	Phthalate esters [59, 93]
	Bisphenol A [59, 93]

Others	Perfluorooctanesulfonic acid (PFOS) [93]
	Perfluoroctanoic acid (PFOA) [93]
	Organochlorine compounds [7]
	Acrylamide [29]
	Dioxins [25, 27]
	Formaldehyde [13–15]
	Methanol [16, 17]
